# Cancer loyalty card study-2 (CLOCS-2): protocol for an observational case-control study focusing on the patient interval in cancer diagnosis

**DOI:** 10.1136/bmjopen-2026-117937

**Published:** 2026-05-13

**Authors:** Sima Toopchiani, Saphsa Codling, Yuscah Pondeca, Sandeep Kaur, Kevin Horeau, Hannah Brewer, Amanda Cross, Brendan Delaney, A Aldo Faisal, Christopher Peters, James Goulding, Yasemin Hirst, Sudha Sundar, James M Flanagan

**Affiliations:** 1 Surgery and Cancer, Imperial College London, London, UK; 2 Department of Computing, Imperial College London, London, UK; 3 Epidemiology and Biostatistics, Imperial College London, London, UK; 4 Department of Surgery and Cancer, Imperial College London, London, UK; 5 Nottingham University Business School, Nottingham, UK; 6 Health Technology Assessment Unit (HTA), University of Central Lancashire, Preston, UK; 7 Cancer Sciences, University of Birmingham, Birmingham, UK

**Keywords:** Early Detection of Cancer, Health informatics, Epidemiology, Clinical Protocols

## Abstract

**Abstract:**

**Introduction:**

Some cancers are diagnosed late, making them harder to treat. People with an undiagnosed cancer may use over-the-counter medications to manage non-specific cancer-related symptoms that often mimic other more common, easily treatable conditions. Results from the original Cancer Loyalty Card Study (CLOCS) suggest there may be an increase in purchases of pain and indigestion medication 8–9 months before an ovarian cancer diagnosis. We aim to validate the CLOCS findings by exploring whether a significant change in medication purchases could be an indication for early signs of the following cancer types: oesophageal, stomach (gastric), colorectal (bowel), pancreatic, liver, bladder, endometrial, uterine sarcoma, ovarian and vulval, using data collected through store loyalty cards.

**Methods and analysis:**

Using a retrospective case-control design, we aim to recruit 1450 participants with one of the cancers of interest (cases) and 1450 participants without cancer (controls) in the UK who (or whose household members) hold a loyalty card with at least one participating high street retailer. We will use pre-existing loyalty card data to compare past purchase patterns of cases with those of controls. To assess cancer risk in participants and their purchasing patterns, we will collect information on demographic characteristics, health risk factors, lifestyle habits and behaviours, family history of cancer and any symptoms experienced prior to diagnosis (cases) and in the last year prior to study recruitment (controls). In addition, cases will be asked about their cancer diagnosis.

**Ethics and dissemination:**

CLOCS-2 was reviewed and approved by the East Midlands-Leicester South Research Ethics Committee (23/EM/0224). Study outcomes will be disseminated through peer-reviewed publications, conferences, presentations to the research communities as well as patients and the public, the study website and other social media outlets.

**Trial registration number:**

NCT06447064, CPMS58679; pre-results.

**STRENGTHS AND LIMITATIONS OF THIS STUDY**

- A key strength is the use of longitudinal purchase data recorded up to 6 years prior to enrolment, enabling analysis of temporal patterns before diagnosis.
- A further strength is the inclusion of multiple cancer types within a single study design, allowing comparison across groups.
- A key limitation is the incomplete capture of purchasing behaviour due to inconsistent loyalty card use and transactions outside participating retailers.
- Another limitation is the potential for exposure misclassification due to reliance on self-reported health survey data among cases and controls.

## Introduction

 Cancer is one of the leading causes of death worldwide, accounting for an estimated 9.6 million deaths each year.^1^ With significant advancements in cancer care and treatment, cancer-related deaths can be reduced when diagnosis and treatment occur at an early stage.^1^ However, for several major cancer types, diagnosis frequently occurs only after the disease has progressed, resulting in a poor prognosis.^2 3^

Early cancer diagnosis is essential to ensure timely treatment and improve patient outcomes.^4^,^6^ Unfortunately, delays can occur at any stage of the diagnostic spectrum/pathway, from the first observation of symptoms to the start of treatment. There are more than 200 types of cancer, each associated with different signs and symptoms. Some symptoms are disease-specific, whereas others are more general, such as weight loss, tiredness (fatigue) or unexplained pain which are often called non-specific cancer symptoms. The type of symptoms experienced can vary between individuals.^4^,^6^

In many cancers, patients may experience vague and/or non-alarming symptoms that resemble common illnesses, which could often seem to resolve or ease with the use of over-the-counter medications and home remedies.^4 7^ As a result, individuals may often self-care for their symptoms or seek advice from other sources such as from pharmacists, family or the internet, rather than from a general practitioner (GP).^8 9^ Common reasons for not presenting to the GP are the perception of “not wanting to waste the GP’s time” or because they have normalised their symptoms,^8 10^ not being able to make an appointment due to the lack of appointments available and looking for information online after not being able to make an appointment.^9^ For example, patients may downplay or normalise their symptoms, attributing them to something harmless, non-threatening or simply accept them as minor or everyday causes linked to their comorbidities.^11^ While persistence or worsening of symptoms, social influences and health literacy can encourage people to seek help from healthcare professionals, few believe their symptom(s) might actually be a sign of cancer until symptoms become persistent or debilitating.^9 12^

While advances in cancer care have been significant, the patient factors associated with earlier presentation to healthcare when individuals are experiencing symptoms are less understood.^11^ The Model of Pathway to Treatment stipulates that an individual’s symptom appraisal involves the evaluation of new symptoms, which is influenced by psychological, social and cultural factors, and may be through self-medication, symptom monitoring and changes in behaviour.^13^ Historically, research on these behaviours has been largely qualitative;^11^ however, more recent studies have begun exploring interventions to promote earlier help-seeking.^14^,^16^

In 2023, the findings from the Cancer Loyalty Card Study (CLOCS) offered a novel observational case-control study approach to test whether purchasing patterns captured through loyalty cards (store reward cards that record customers’ purchase histories) could provide early warning signals of ovarian cancer.^17^ The results showed that, compared with participants without ovarian cancer, those with ovarian cancer had a significant increase in purchases of indigestion and pain medications up to 9 months before a diagnosis of ovarian cancer was received (OR 1.38, 95% CI 1.04 to 1.83, p=0.03).^17^

Ovarian cancer was chosen to be the first type of cancer to be explored due to its non-specific symptoms and patient-reported experiences using the over-the-counter medication. Building on the findings of the original CLOCS, CLOCS-2 aims to validate these findings across ovarian cancer as well as nine additional cancers: oesophageal, stomach (gastric), colorectal (bowel), pancreatic, liver, bladder, endometrial, uterine sarcoma, ovarian and vulval cancers. These cancer types have been identified as cancers in which patients experience non-specific symptoms and self-care for^11^ and with highest gains in patient treatment outcomes and survival if diagnosed early.

A phased approach will be used. Four cancers (oesophageal, colorectal (bowel), pancreatic and ovarian), for which symptom profiles and patient pathways are better characterised, will be prioritised for full evaluation. The remaining six cancers will initially be piloted, as there is greater uncertainty regarding symptom presentation and the feasibility of recruitment within these groups. The pilot phase will inform the feasibility of wider inclusion of these cancers in the study.

If successful this study may lead to future interventions that might include sending an alert to individuals who exceed the ‘alert threshold’, advising them to visit a GP to discuss their symptoms, or delivering a potential public health message about purchase thresholds and the risks of self-treating potential cancer symptoms.

## Study objectives

The primary objective of this case-control study is to identify the time points at which participants with the following cancers (cases): oesophageal, stomach (gastric), colorectal (bowel), pancreatic, liver, bladder, endometrial, uterine sarcoma, ovarian and vulval, show statistically significant differences in their purchasing behaviours compared with purchase behaviours of participants without a cancer diagnosis (controls) over the previous 6 years. For controls, the purchases included are those recorded from participant consent to the study, up to a maximum of 6 years prior. For cases, the purchases included are those recorded up to the date of cancer diagnosis, to a maximum of 6 years, depending on when the diagnosis occurred. These behaviours include the purchase of items suspected of being used for symptom management (eg, painkillers, indigestion tablets) as well as changes in the frequency or quantity of other items purchased leading up to diagnosis. Participants’ historic loyalty card data will be collected from two major high street retailers.

The secondary objectives of CLOCS-2 are to: (1) Define a purchase threshold that may act as an “alert” for potential cancer symptoms in individuals and assess the predictive value of purchasing behaviours in the early detection of these cancers, (2) Develop a predictive model to determine the accuracy and efficacy of these purchasing behaviours for early cancer detection and (3) Create risk profiles from the participants’ self-reported health data and incorporate these into the analyses.

## Methods and statistical analysis

We have completed the Strengthening the Reporting of Observational Studies in Epidemiology checklist, which is provided as online supplemental material S1.

### Study design and setting

CLOCS-2 will use an observational retrospective case-control design to compare past purchase patterns of participants (men and women) with and without a cancer diagnosis using already existing data gathered by two major high street retailers, one of which is a health and beauty retailer (Boots), and the other a groceries and general merchandise retailer (Tesco). Furthermore, we will collect data on risk factors for the following cancers: oesophageal, stomach (gastric), colorectal (bowel), pancreatic, liver, bladder, endometrial, uterine sarcoma, ovarian and vulval.

CLOCS-2 is a secure online study delivered via Research Electronic Data Capture (REDCap), a web-based application designed to support data capture for research studies, providing secure data storage, audit trails and easy survey administration.^18^

The study documents, including the informed consent form, patient information sheet (PIS), and the health survey, are available in English, Gujarati and Polish. This represents an improvement over the original CLOCS study. Gujarati and Polish were selected as additional languages because they were the most commonly spoken within the relevant Research Delivery Networks (RDNs) and Primary Care Networks (PCNs) in the study area.

### Study timeline

CLOCS-2 study recruitment is planned to take place from 4 February 2026 until January 2028.

### Participants

#### Inclusion/exclusion criteria

**Figure 1:**
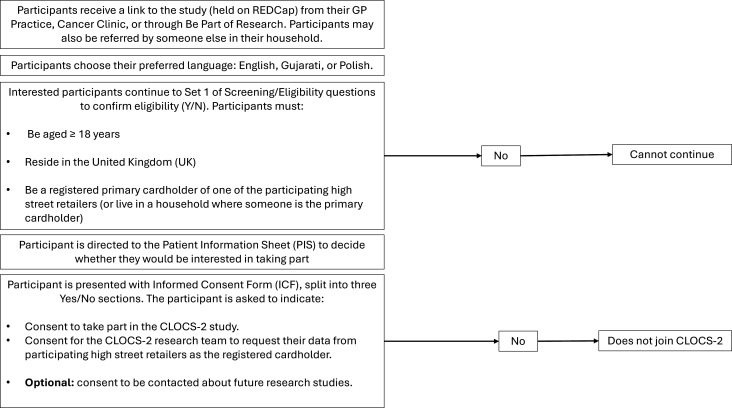
Cancer Loyalty Card Study-2 (CLOCS-2) recruitment and consent process. Overview of participant identification, invitation, eligibility screening and informed consent. GP, general practitioner; REDCap, Research Electronic Data Capture.

The CLOCS-2 will recruit up to 2900 people for the study. This will comprise two participant groups: cases (n=1450) and controls (n=1450). The eligibility criteria for both participant groups (men and women) are that participants must be at least 18 years old, reside in the UK at the time of giving informed consent, and be registered with an NHS GP practice. Participants must own, or have a household member who owns, at least one participating high street retailer loyalty card to be eligible to join the CLOCS-2 case-control study. Finally, all participants must be able to provide written informed consent and willingness to comply with all required study activities (figure 1).

#### Eligibility for cases

Men and women diagnosed and living with an eligible cancer type within the 2 years prior to the recruitment date are eligible to join the CLOCS-2 case-control study as cases. Patients’ usual care will continue to be provided by their usual healthcare professionals, and CLOCS-2 participation does not exclude patients from participating in other clinical or non-clinical studies.

#### Eligibility for controls

Men and women who have not been diagnosed with any cancer in the last 6 years prior to recruitment (except where the diagnosis was non-melanoma skin cancer).

### Recruitment

The CLOCS-2 case-control study recruitment and consent process is outlined in figure 1 and described further below.

Participants (cases and controls) will be recruited across the UK through individual GP practices and Be Part of Research. Additional cases will be recruited through NHS cancer c*linics.*

#### GP practices

After submitting an expression of interest, new RDNs or PCNs will receive further study information and set up an organisation information document (OID) for each participating GP practice. The OID developed with the North London RDN will serve as a template for other networks. A brief site initiation visit (SIV) will be held with each new RDN/PCN conducted by the CLOCS-2 team.

Clinical team members at each GP practice will identify potential study participants with a primary diagnosis of one of the eligible cancers, received in the last 2 years prior to recruitment date. Eligible cases will be randomly selected if the number of potential participants exceeds the required size. GP practices will match invited cases with eligible controls based on age bracket, sex and the number of inhabitants. Controls will be randomly sampled from participants without a cancer diagnosis who meet the matching criteria, typically selecting one control per case. Once eligible participants are identified, the GP practice will send a text message (drafted by the CLOCS-2 team).

#### Be part of research

Be Part of Research is a UK-based participant recruitment service run by the National Institute for Health and Care Research, which is open to anyone living in the UK. Registered volunteers provide demographic and health information and can indicate the types of studies they are interested in. The platform then pre-screens individuals based on this information and their stated interests to identify those who may be eligible for specific studies. Individuals who meet these criteria may be contacted by email with information about the CLOCS-2. All respondents will undergo further screening against the study’s inclusion and exclusion criteria prior to enrolment.

#### NHS cancer clinics

Recruitment may also take place at NHS cancer clinics if we are unable to meet our recruitment target for cases. An OID will be created for each recruited clinic in the same way as the RDNs/PCNs. Capacity and capability will be sought from each cancer clinic, with an SIV held prior to recruitment. Cancer clinics will follow the same procedures as the GP practices, that is, identify potential study participants with a primary diagnosis of one of the eligible cancer types received in the last 24 months. As with GP practices, the only way a person can access our recruitment site is via a link received by a text message.

Eligible patients with a cancer diagnosis from one of the ten eligible cancers will be recruited by members of their healthcare teams at selected NHS trusts and hospitals across the UK that have agreed to approach patients at clinics. These healthcare teams will be registered as a research site and will have a principal investigator (PI) leading the recruitment to CLOCS-2. The PI and the research nurses will facilitate recruitment by identifying patients (cases) when they think it is suitable to approach patients with the study information to consider participation.

### Informed consent

Both cases and controls will need to complete a digital consent form to participate in the CLOCS-2. The consent form includes three sections. The first section details consent to take part in CLOCS-2. The second section is for participants to consent to the research team requesting their data from high street retailers as the registered keeper. The third section is optional and relates to participation in future research. Participants who are not the primary registered card holder but share the use of the loyalty card or live in a household where the primary cardholder makes purchases on their behalf are still eligible to take part in the study. However, to access their shared loyalty data, the primary registered cardholder must also register for the study. If the primary cardholder does not join the study, the CLOCS team will not request shopping data from the high street retailer and will use only the data from the health survey they completed.

All individual consents will be transferred from REDCap to the secure and encrypted server within Imperial College London’s Certified Secure Research Environment (CSRE), formerly known as the ‘Secure Enclave’, once study recruitment is complete. Participant loyalty card details will be entered into a comma-separated values (CSV) file and encrypted for secure data requests from retailers using a secure file transfer protocol. After recruitment is finished and all transfers are complete, individual consent forms will be permanently deleted from REDCap.

### Data sources and variables

#### Loyalty card data (for cases and controls)

CLOCS-2 participants or someone in their household will hold a loyalty card at one or both participating retailers that can be accessed by the UK population within all precincts, allowing the study to assess purchases of over-the-counter medications (ie, painkillers and indigestion medications) and eating habits. Eating habits will be used to identify dietary risk factors for specific cancers and to examine changes in dietary behaviour in response to potential cancer symptoms. The study will not include prescription-based pharmacy data which are not recorded in loyalty cards. After participants have provided consent, the CLOCS-2 team or the participant through the retailers’ OpenID platform (an interoperable authentication protocol that simplifies the way to verify the identity of users) will request their loyalty card data from the retailer, which will include individuals’ past purchase records from up to 6 years prior to the relevant cut-off date (study consent for controls or date of cancer diagnosis for cases). The purchase data will include each individual purchase, date of purchase, the location (ie, store postcode) and the product categorisation that is provided by the retailers. No additional individual information will be requested from the retailers.

### Health survey

**Figure 2:**
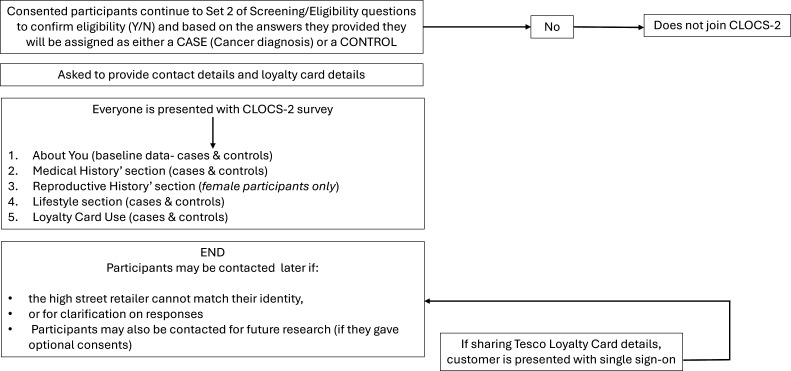
CLOCS-2 questionnaire flow following informed consent. Flow diagram showing the sequence of the online questionnaire completed after consent including sections on health status, medical history and lifestyle factors and the approximate duration of each section. CLOCS-2, Cancer Loyalty Card Study-2.

After completing consent, participants will be required to complete an online questionnaire (approximately 50 min) about their health, medical history and lifestyle (figure 2). The design of this questionnaire has been informed by our understanding of well-established cancer risk factors and symptoms reported by patients across the target cancers in previous research.^11^ Participants with a cancer diagnosis will also be asked to provide details on the cancer type (oesophageal, stomach (gastric), colorectal (bowel), pancreatic, liver, bladder, endometrial, uterine sarcoma, ovarian and vulval) and stage (I, II, III and IV) of cancer. We will use this information to inform risk assessment and to stratify loyalty card purchasing data for cases and controls by time of diagnosis. Purchasing patterns will then be examined by cancer type and stage at diagnoses.

### Data extraction from the high street retailers

For both high street retailers, we will request up to 6 years in total of back-dated purchase data. The exact amount of data available for each participant will depend on how long they have had their loyalty card. Following participant consent, this data will be backdated from the date of study consent for controls or from the date of cancer diagnosis for cases.

**Figure 3:**
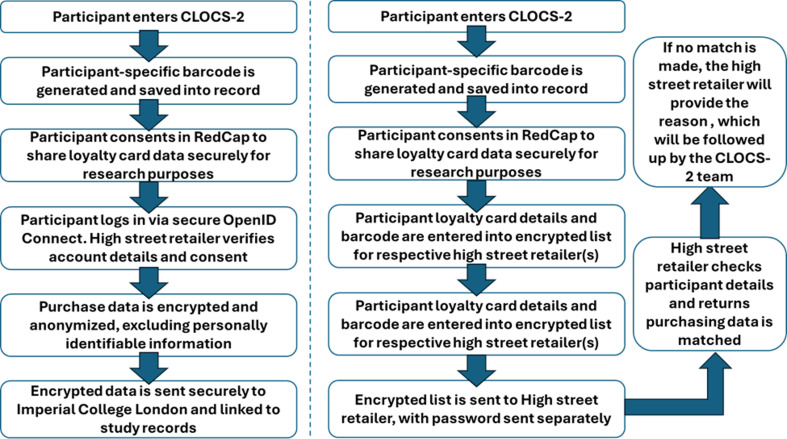
Automated and manual process for obtaining loyalty card purchasing data from high street retailer(s) (a) Automated process showing secure data transfer between retailer (Tesco) and the CLOCS-2 research team following participant consent. (b) Manual process illustrating participant-mediated data sharing, including data request, download and submission steps.

The process for requesting data will differ between the two participating high street retailers. For Tesco, we will also use their Open ID Connect platform (figure 3). To facilitate this, a custom landing page was built which directs participants from REDCap to the retailer’s OpenID platform where participants will be able to sign in using their store login details. This ensures the retailer receives direct consent from the participants to share their loyalty card data with the CLOCS-2 research team. This approach eliminates the need for manual identity verification (eg, uploading ID documents), which was a barrier in the original CLOCS. It streamlines the process, reduces participant burden and improves data security by allowing the retailer to authenticate participants directly. It also ensures compliance with the retailer policies for sharing purchase data with third parties.

For both participating retailers, a barcode ID will be generated for each primary registered cardholder by the CLOCS-2 team prior to transfer from Imperial College London to the high street retailer (figure 3). We will ask participants to provide their full name (as written on the loyalty card) and loyalty card number. The CLOCS-2 team will send the CSV file that contains a unique study ID, name of the individual and the exact loyalty card number specific to the retailer using the secure file transfer. The respective high street retailers will validate the participant information from the consent form and their loyalty card data from the health questionnaire. High street retailers will then centrally generate a dataset for all consenting individuals, in accordance with data sharing agreements, and will share participants’ past purchase data with the CLOCS-2 team for the period prior to the date of recruitment.

### Data merge

The CLOCS-2 team will receive the encrypted participant purchase history from the high street retailers through the SFTP, save and store all data to a secure and encrypted server based on the CSRE at Imperial College London, described in the Data Management section. All data will be pseudonymised and analysed on the secure server.

### Data deletion

The information relating to the participants’ consents to providing past purchase data will be retained in line with each high street retailer’s data retention policy. Ten years after the study is complete, all paper-based and electronically held identifiable data including the electronic consent forms will be destroyed or deleted in accordance with Imperial College London’s data deletion and retention processes.

Contact details will be kept in REDCap at Imperial College London only for project management and facilitation purposes while the project is actively recruiting and participant data is stored on REDCap. Once the data has been fully transferred from REDCap to the CSRE, contact details will be permanently deleted unless participants have authorised us to retain them for follow-up research or to receive study results once published.

Past purchase loyalty card data and health data (as entered by participants through REDCap) will be kept pseudonymised either until all the results are published or until 5 years after the study has completed, whichever comes first. Then all past purchase data and health data will be fully anonymised and any link to identifiable data deleted.

### Sample size and statistical power

A power calculation was conducted for the following four cancers: ovarian, pancreatic, oesophageal and colorectal. This calculation was based on the former CLOCS study^19^ which was conducted using conditional logistic regression of matched pairs. The calculation assumes an effect size of OR=1.38, with 80% power at alpha=0.05 and estimates n=223 case-control pairs are needed for each cancer type. To allow for attrition of ~10%, the total number we will aim to recruit for each of these four cancers will be 250 (n=1000). No power calculation has been done for the remaining six types of cancer as these are being piloted, but the aim is to recruit 75 participants per cancer.

### Statistical methods

We will use descriptive statistics to provide details on the characteristics of cases and controls by sociodemographic (eg, age, sex and ethnicity), cancer type and by risk factors.^11^ We will report feasibility factors, including the number of participants with a loyalty card from either participating retailer, the number with cards from both retailers and the number of years of data available for analysis.

We will aim to define the time by which cases and controls become statistically significantly different in their purchase behaviours using a multivariable, conditional logistic regression model with cancer as the outcome and ‘purchase proportion’ at different timepoints to diagnosis as the exposure, adjusting for potentially confounding variables. This analysis will be repeated and stratified by cancer type, stage at diagnosis and sociodemographic factors. Analyses will be performed for different types of medications and cancers separately and combined.

We will compare proportions using Fisher’s exact tests comparing target (items suspected to be used to treat cancer symptoms) and non-target (all other items) purchases. These comparisons will be made between cases and controls at each month prior to diagnosis. For controls, the reference will be the average monthly proportions of purchases across the observation period. Target items for analysis will include, but will not be limited to, medications for pain, indigestion, bloating relief and fatigue, along with other items commonly used to alleviate cancer-related and non-specific symptoms.^11^

We will conduct exploratory analyses to establish a potential risk profile for each participant and cancer type. This will be defined as a mathematical summation of all relevant risk factors, based on the published literature.^11^ In line with the findings from the original CLOCS, this analysis will be exploratory.^19^ Furthermore, we will investigate additional methods that leverage autocorrelation in the longitudinal data. Specifically, we will examine the evolution of individual purchasing frequencies and purchase trajectories to identify patterns—such as increases in purchases prior to diagnosis or distinct purchase peaks—that may be indicative or predictive of disease onset and time to onset.

All analyses will be done using statistical software R.

### Interim analysis

An interim analysis will be conducted after 6 months of CLOCS-2 recruitment to provide an initial exploratory analysis of the data and assess recruitment and the demographics of CLOCS-2 participants. This will help determine whether recruitment efforts need to be re-focused to target specific demographics (eg, age groups and sex) or cancer types to ensure a representative distribution of cases and controls for the matched analysis.

### Study risks and sources of bias

There are no known risks to participants in the CLOCS-2 study, in line with the original CLOCS.^19^ However, some participants may find it emotionally difficult to think about cancer or to recall when they first noticed bodily changes (ie, symptoms). The CLOCS-2 team has carefully designed the survey to be sensitive and respectful, aiming to minimise any potential discomfort and to protect participants’ well-being, with input from patient and public representatives during the design of the study and the health survey. Participants are advised, via the PIS, to consult a healthcare professional if they have any concerns about any symptoms or signs they may be experiencing.

The key limitations of the CLOCS-2 are consistent with those identified in the original CLOCS.^19^ As noted previously, loyalty card data may not capture all relevant purchases, as participants may not use their loyalty cards for every transaction and may shop at other retail stores not included in this study. Additionally, purchases may be made for other household members, making it difficult to determine precisely which items were consumed by the participant. However, reasonable assumptions will be made to account for household purchasing patterns in the analysis including information collected through the health survey (eg, frequency of loyalty card use, primary shopping locations and comorbidities). Finally, as all study procedures (including consent, questionnaire completion and submission of loyalty card details) are conducted digitally, individuals with limited digital skills may find it difficult to participate. While some participants may be able to receive assistance, those without access to such support may be less likely to take part, which could introduce selection bias.

The original CLOCS protocol also recognised and outlined that some over-the-counter purchases may relate to other comorbidities rather than to symptoms of cancer. As prescribed medications are not captured within loyalty card data, we will interpret findings cautiously and adjust for relevant self-reported comorbidities and GP visit frequency. The generalisability of the study results may also be limited to populations with loyalty cards from participating retailers, and differences in demographics, healthcare systems or purchasing behaviours may affect applicability to other populations.

### Data management

The CLOCS-2 will follow the data management framework noted in the original CLOCS,^19^ in which data processing and storage will adhere to strict confidentiality and security standards. Under this framework, the CLOCS-2 study team will act as data processors when handling retailer data and as data controllers for self-reported and health survey data. The high street retailers would be data controllers of the shopping data. All participant data will be pseudonymised, stored in Imperial College’s ISO27001-CSRE and accessible only to the research team. A rigorous data protection model has been established to ensure security of personal and medical data. The consenting participants will be data owners, data handling complies with GDPR and institutional data governance procedures, ensuring participants’ right to withdraw consent at any time.

The study website complies with Imperial College London’s data security standards and GDPR, employing public key encryption to ensure that all online survey data reside solely within the CSRE. The platform is also designed to be user-friendly and accessible to mobile users.

Strict data processing agreements are maintained with commercial partners to guarantee ongoing data protection, and these partners will not have access to any research data and will not receive financial benefit from the study.

### Public, patient involvement and engagement (PPIE)

The original CLOCS was supported by two ovarian cancer patients and other patient representatives. For CLOCS-2, further meetings were held, and we have gained further support from patient advocates representing colorectal (bowel), oesophageal, pancreatic and ovarian cancer.

Multiple public engagement activities have been carried out, accompanied by extensive media coverage of the original CLOCS findings. We have also held public meetings at the Maggie’s Centre (London), Imperial Lates, the Imperial Great Exhibition Road Festival and the Parklife Cafe (Harrow Weald). Public engagement efforts will continue throughout the duration of the CLOCS-2 study, with activities focused on reducing participation barriers and increasing public awareness of the study. These activities will include annual scientific meetings (involving patient advocates), public webinars and targeted work with other community organisations. Co-design workshops will also be conducted with patient representatives and other key stakeholders. Collectively, these activities will help us determine how candidate “early cancer symptom” alert mechanisms could be implemented and inform design of a future intervention study.

### Ethics

CLOCS-2 was reviewed and approved by the East Midlands-Leicester South Research Ethics Committee (Ref: 23/EM/0224). The study is registered as an NIHR portfolio study and is registered on ClinicalTrials.gov. This paper describes the ethics-approved protocol dated 18 July 2025 (V.5.0).

### Data Sharing Policy

Access to CLOCS-2 data collected from retailers will be strictly restricted and will not be shared with external researchers, in accordance with the data sharing agreements with the retailers. However, researchers interested in collaboration are encouraged to contact the CLOCS-2 team. Where appropriate, they may be granted the opportunity to review metadata after contacting the team and submitting a research proposal. Proposals will be reviewed on a case-by-case basis by the Principal Investigator (PI) and the research team. All data analyses will be conducted by a member of the CLOCS-2 team.

## Supplementary material

10.1136/bmjopen-2026-117937 online supplemental file 1

## References

[1] Coleman MP, Forman D (2011). Cancer survival in Australia. Lancet.

[2] Schiffman JD, Fisher PG, Gibbs P (2015). Early detection of cancer: past, present, and future. Am Soc Clin Oncol Educ Book.

[3] Crosby D, Bhatia S, Brindle KM (2022). Early detection of cancer. Science.

[4] Ott JJ, Ullrich A, Miller AB (2009). The importance of early symptom recognition in the context of early detection and cancer survival. Eur J Cancer.

[5] Whitaker K (2020). Earlier diagnosis: the importance of cancer symptoms. Lancet Oncol.

[6] Koo MM, Swann R, McPhail S (2020). Presenting symptoms of cancer and stage at diagnosis: evidence from a cross-sectional, population-based study. Lancet Oncol.

[7] Whitaker KL, Winstanley K, Macleod U (2015). Low cancer suspicion following experience of a cancer “warning sign”. Eur J Cancer.

[8] Robinson D, Reguilon I (2016). International Cancer Benchmarking Partnership, Evidence for policy and practice.

[9] Whitelock V (2024). Cancer Research UK’s 2024 Cancer Awareness Measure ‘Plus’ (CAM+).. https://www.cancerresearchuk.org/health-professional/awareness-and-prevention/the-cancer-awareness-measures-cam-plus/archive?utm_source=chatgpt.com.

[10] Low EL, Whitaker KL, Simon AE (2015). Women’s interpretation of and responses to potential gynaecological cancer symptoms: a qualitative interview study. BMJ Open.

[11] Wilson G, Brewer HR, Flanagan JM (2024). How Do Patients Use Self-Care to Manage Nonspecific Symptoms Prior to a Cancer Diagnosis? A Rapid Review to Inform Future Interventions to Reduce Delays in Presentation to Primary Care. Eur J Cancer Care (Engl).

[12] Whitaker KL, Macleod U, Winstanley K (2015). Help seeking for cancer “alarm” symptoms: a qualitative interview study of primary care patients in the UK. Br J Gen Pract.

[13] Scott SE, Walter FM, Webster A (2013). The model of pathways to treatment: conceptualization and integration with existing theory. Br J Health Psychol.

[14] Wagland R, Brindle L, Ewings S (2016). Promoting Help-Seeking in Response to Symptoms amongst Primary Care Patients at High Risk of Lung Cancer: A Mixed Method Study. PLoS ONE.

[15] Smits S, McCutchan G, Wood F (2018). Development of a Behavior Change Intervention to Encourage Timely Cancer Symptom Presentation Among People Living in Deprived Communities Using the Behavior Change Wheel. Ann Behav Med.

[16] Saab MM, FitzGerald S, Noonan B (2021). Promoting lung cancer awareness, help-seeking and early detection: a systematic review of interventions. Health Promot Int.

[17] Brewer HR, Hirst Y, Chadeau-Hyam M (2023). Association Between Purchase of Over-the-Counter Medications and Ovarian Cancer Diagnosis in the Cancer Loyalty Card Study (CLOCS): Observational Case-Control Study. JMIR Public Health Surveill.

[18] Kragelund SH, Kjærsgaard M, Jensen-Fangel S (2018). Research Electronic Data Capture (REDCap®) used as an audit tool with a built-in database. J Biomed Inform.

[19] Brewer HR, Hirst Y, Sundar S (2020). Cancer Loyalty Card Study (CLOCS): protocol for an observational case-control study focusing on the patient interval in ovarian cancer diagnosis. BMJ Open.

