# Supplementary material for: Cancer loyalty card study-2 (CLOCS-2): protocol for an observational case-control study focusing on the patient interval in cancer diagnosis
Source: BMJ Open. 2026 May 13;16(5):e117937. doi: 10.1136/bmjopen-2026-117937 (PMC13182332; doi:10.1136/bmjopen-2026-117937)
Supplement: online supplemental file 1 [file bmjopen-16-5-s001.docx]

**Supplementary**

Supplementary Table S1: STROBE Checklist for Observational Study Reporting

|  | Item No. | Recommendation | Page  No. | Relevant text from manuscript |
| --- | --- | --- | --- | --- |
| **Title and abstract** | 1 | (*a*) Indicate the study’s design with a commonly used term in the title or the abstract | 1 | Cancer Loyalty Card Study-2 (CLOCS-2): protocol for an observational case-control study focusing on the patient interval in cancer diagnosis. |
|  |  | (*b*) Provide in the abstract an informative and balanced summary of what was done and what was found | 2 | This protocol describes a retrospective case-control study. Purchase data from loyalty card’s data from two high-street retailers were used as well as data collected via a health questionnaire. |
| Introduction | | | |  |
| Background/rationale | 2 | Explain the scientific background and rationale for the investigation being reported | 4 | For several major cancer types, diagnosis frequently occurs only after the disease has progressed, resulting in a poor prognosis. For many of these cancers, patients may experience non-symptomatic or vague symptoms that often seem easy to resolve with over-the-counter medications. Findings from the Cancer Loyalty Card Study (CLOCS) showed that participants with ovarian cancer were more likely to buy indigestion and pain medications up to nine months before a cancer diagnosis. |
| Objectives | 3 | State specific objectives, including any prespecified hypotheses | 6 | The primary objective of this case-control study is to identify, the time points at which participants with specific cancers (cases) show statistically significant differences in their purchasing behaviours compared with purchase behaviours of participants without a cancer diagnosis (controls).  Secondary objectives include i) defining a purchase threshold that may act as an “alert” for potential cancer symptoms, ii) developing a predictive model to determine the accuracy and efficacy of these purchasing behaviours for early cancer detection, and iii) creating risk profiles from the participants’ self-reported health data and incorporate these into the analyses. |
| Methods | | | |  |
| Study design | 4 | Present key elements of study design early in the paper | 6 | This study uses an observational retrospective case-control design to compare purchase patterns of participants with and without cancer diagnosis. |
| Setting | 5 | Describe the setting, locations, and relevant dates, including periods of recruitment, exposure, follow-up, and data collection | 6 & 7 | Data will be collected using already existing data gathered by two major high street retailers up to a maximum of six years. Data will also be collected on risk factors for the specific cancers via an online health questionnaire delivered via REDCap. The CLOCS-2 study is planning to recruit from 4^th^ February 2026 until January 2028. |
| Participants | 6 | (*a*) *Cohort study*—Give the eligibility criteria, and the sources and methods of selection of participants. Describe methods of follow-up  *Case-control study*—Give the eligibility criteria, and the sources and methods of case ascertainment and control selection. Give the rationale for the choice of cases and controls  *Cross-sectional study*—Give the eligibility criteria, and the sources and methods of selection of participants | 7 | The CLOCS-2 will recruit up to 2,900 participants. This will comprise two participant groups: cases (n=1,450) and controls (n=1,450). The eligibility criteria for both participants groups (men and women) are they must be adults. Registers with an NHS GP practice, must own at least one participating high street retailers’ loyalty card and must be able to provide written informed consent and willingness to comply with all required study activities. Differences in eligibility include a diagnosis of an eligible cancer type in the last 6 years prior to recruitment and for controls there would be no diagnosis of cancer in the last 6 years prior to recruitment. |
|  |  | (*b*) *Cohort study*—For matched studies, give matching criteria and number of exposed and unexposed  *Case-control study*—For matched studies, give matching criteria and the number of controls per case | 9 | Matching will occur from participants without a cancer diagnosis (controls) will be matched on age bracket, sex, and the number of inhabitants, typically selecting one control per case. |
| Variables | 7 | Clearly define all outcomes, exposures, predictors, potential confounders, and effect modifiers. Give diagnostic criteria, if applicable |  | Outcomes for cases include a diagnosis of |
| Data sources/ measurement | 8* | For each variable of interest, give sources of data and details of methods of assessment (measurement). Describe comparability of assessment methods if there is more than one group | 10 | Data from existing purchase data from loyalty cards will include each individual purchase, date of purchase, the location (i.e. store postcode) and the product categorization that is provided by the retailers.  After completing consent, participants will be required to complete an online questionnaire about their health, medical history, and lifestyle. Participants with cancer (cases) will also be asked to provide details on the cancer type and stage of cancer. |
| Bias | 9 | Describe any efforts to address potential sources of bias | 16 | Some participants may find it emotionally difficult to this about cancer or recall symptoms. The CLOCS-2 team has carefully designed the survey to be sensitive and respectful, minimising potential discomfort. Additionally, as purchases may be made for other household members, however, reasonable assumptions will be made to account for household purchasing patterns. |
| Study size | 10 | Explain how the study size was arrived at | 14 | A power calculation was conducted for the following four cancers: ovarian, pancreatic, oesophageal, and colorectal. The calculation was based on the original CLOCS study. The calculation was based on an effect size of OR=1.38, with 80% power at alpha=0.05 and attrition of ~10%. |

Continued on next page

| Quantitative variables | 11 | Explain how quantitative variables were handled in the analyses. If applicable, describe which groupings were chosen and why | 15 | Quantitative variables with cancer as the outcome (will be treated as categorical) and ‘purchase proportion’ at different timepoints to diagnosis as the exposure. This analysis will be repeated and stratified by cancer type, stage at diagnosis, and sociodemographic factors. |
| --- | --- | --- | --- | --- |
| Statistical methods | 12 | (*a*) Describe all statistical methods, including those used to control for confounding | 15 | In the study analyses we will adjust for potential confounders. |
|  |  | (*b*) Describe any methods used to examine subgroups and interactions | 15 | We will use Fisher’s exact tests to compare target and non-target purchases. These comparisons will be made between cases and controls at each months prior to diagnosis. |
|  |  | (*c*) Explain how missing data were addressed |  | Not applicable |
|  |  | (*d*) *Cohort study*—If applicable, explain how loss to follow-up was addressed  *Case-control study*—If applicable, explain how matching of cases and controls was addressed  *Cross-sectional study*—If applicable, describe analytical methods taking account of sampling strategy |  | Not applicable |
|  |  | (*e*) Describe any sensitivity analyses |  | Exploratory |
| Results | | | | |
| Participants | 13* | (a) Report numbers of individuals at each stage of study—eg numbers potentially eligible, examined for eligibility, confirmed eligible, included in the study, completing follow-up, and analysed | Methods | As this is a protocol, no participants have been enrolled yet. Once conducted, we will report numbers at each stage. |
|  |  | (b) Give reasons for non-participation at each stage | Methods | Reasons for non-participation (e.g., refusal, withdrawal) will be documented and reported in the final study. |
|  |  | (c) Consider use of a flow diagram | Methods | A flow diagram illustrating participant flow will be provided in the final report. |
| Descriptive data | 14* | (a) Give characteristics of study participants (eg demographic, clinical, social) and information on exposures and potential confounders | Results | In the final report we will provide details on the characteristics of cases and controls. |
|  |  | (b) Indicate number of participants with missing data for each variable of interest | Methods | A table showing the number of participants with missing data for each variable of interest will be provided in the final report. |
|  |  | (c) *Cohort study*—Summarise follow-up time (eg, average and total amount) |  | Not applicable |
| Outcome data | 15* | *Cohort study*—Report numbers of outcome events or summary measures over time |  | Not applicable |
|  |  | *Case-control study—*Report numbers in each exposure category, or summary measures of exposure | Results | Once recruitment and data analysis has been completed, results will be provided in the final report. |
|  |  | *Cross-sectional study—*Report numbers of outcome events or summary measures |  |  |
| Main results | 16 | (*a*) Give unadjusted estimates and, if applicable, confounder-adjusted estimates and their precision (eg, 95% confidence interval). Make clear which confounders were adjusted for and why they were included | Results | Will be provided in the final report following data analysis. |
|  |  | (*b*) Report category boundaries when continuous variables were categorized | Results | Will be provided in the final report following data analysis. |
|  |  | (*c*) If relevant, consider translating estimates of relative risk into absolute risk for a meaningful time period | Results | Will be provided in the final report following data analysis. |

Continued on next page

| Other analyses | 17 | Report other analyses done—eg analyses of subgroups and interactions, and sensitivity analyses | Results | Exploratory analyses will be conducted to establish a potential risk profile for each participant and cancer type. These results will be reported in the final report. |
| --- | --- | --- | --- | --- |
| Discussion | | | | |
| Key results | 18 | Summarise key results with reference to study objectives | Discussion | Following study completion and data analysis, a summary of results will be reported in the final report. |
| Limitations | 19 | Discuss limitations of the study, taking into account sources of potential bias or imprecision. Discuss both direction and magnitude of any potential bias | 16 | Limitations of the study include loyalty card data that may not capture all relevant purchases, or purchases that may be made for other household members. Additionally, as all study procedures are conducted digitally, individuals with limited digital skills and assistant may find it difficult to participate, which could introduce selection bias. |
| Interpretation | 20 | Give a cautious overall interpretation of results considering objectives, limitations, multiplicity of analyses, results from similar studies, and other relevant evidence | Discussion |  |
| Generalisability | 21 | Discuss the generalisability (external validity) of the study results | 17 | The generalisability of the study results may be limited to populations with loyalty cards from participating retailers, and differences in demographics, healthcare systems or purchasing behaviours may affect applicability to other populations. |
| Other information | |  | | |
| Funding | 22 | Give the source of funding and the role of the funders for the present study and, if applicable, for the original study on which the present article is based | 21 | The CLOCS-2 study was supported by Cancer Research UK (CRUK). |

*Give information separately for cases and controls in case-control studies and, if applicable, for exposed and unexposed groups in cohort and cross-sectional studies.

**Note:** An Explanation and Elaboration article discusses each checklist item and gives methodological background and published examples of transparent reporting. The STROBE checklist is best used in conjunction with this article (freely available on the Web sites of PLoS Medicine at http://www.plosmedicine.org/, Annals of Internal Medicine at http://www.annals.org/, and Epidemiology at http://www.epidem.com/). Information on the STROBE Initiative is available at www.strobe-statement.org.
