# Supplementary material for: Cancer loyalty card study-2 (CLOCS-2): protocol for an observational case-control study focusing on the patient interval in cancer diagnosis
Source: BMJ Open. 2026 May 13;16(5):e117937. doi: 10.1136/bmjopen-2026-117937 (PMC13182332; doi:10.1136/bmjopen-2026-117937)
Supplement: Supplementary file 2 [file bmjopen-2026-117937.reviewer_comments.pdf]

PEER REVIEW HISTORY

BMJ Open publishes all reviews undertaken for accepted manuscripts. Reviewers are asked to complete a checklist review form and are provided with free text boxes to elaborate on their assessment. These free text comments are reproduced below.

ARTICLE DETAILS

Title (Provisional)

Cancer Loyalty Card Study-2 (CLOCS-2): protocol for an observational case-control study focusing on the patient interval in cancer diagnosis

Authors

Toopchiani, Sima; Codling, Saphsa; Pondeca, Yuscah; Kaur, Sandeep; Horeau, Kevin; Brewer, Hannah; Cross, Amanda; Delaney, Brendan; Faisal, A. Aldo; Peters, Christopher; Goulding, James; Hirst, Yasemin; Sundar, Sudha; Flanagan, James

---

VERSION 1 - REVIEW

**Reviewer**	**1**
**Name**	**Thulesius, Hans**
**Affiliation**	**Lund University**
**Date**	**12-Mar-2026**
**COI**	**None**

The only thing I miss in this protocol paper is the use of STROBE and/or RECORD guidelines checklist.

**Reviewer**	**2**
**Name**	**Brekke, Mette**
**Affiliation**	**University of Oslo, Institute of Health and Society**
**Date**	**19-Mar-2026**
**COI**	**None**

Thank you for the opportunity to review the protocol for this interesting, elaborate and well-planned study.

I have focused on the presentation of the protocol, not on the design of the study as such. That means: Is the protocol clear? Is something missing? Is the rationale for the study clearly described?

This is a complex and elaborate study, involving retailers, GPs and other healthcare providers, as well as patients and healthy controls. It builds on a similar, but smaller study which the research team has already carried out. All in all, the protocol describes the study well – the rationale, the aim(s), the methods, the analyses, strengths and weaknesses. The dates for the study are included – recruitment of participants started in February 2026 (as the study is not dependent upon the publication of the protocol). Acceptance from Ethics committee as well as funding sources are revealed.

My comments below mainly refer to questions around some unclarities:

1. You aim to recruit 1 450 participants with one of the cancers of interest (cases) and 1 450 participants without cancer (controls). But you also say: "Controls will be randomly sampled from patients without a cancer diagnosis who meet the matching criteria, typically selecting multiple controls per case…" How does this relate to the consistent numbers of 1 450 in each group?

2. In the framed description of strengths and limitations on p. 4 you say: …" investigate whether routinely collected commercial purchase data can be used to reduce delayed diagnosis of the following cancers…". This may be your ultimate goal, however it is not the aim of the present study, even if one of your secondary aims is to develop theoretical models based upon the results. Further down (page 6) you write: "If successful this study may lead to future interventions…". This seems to be the correct way of presenting your hopes. To show reduced delayed diagnoses would require an intervention study.

3. This comment relates to the one above. On p. 7 you describe your secondary aims and write that one of these are to "Develop and evaluate a predictive model to determine the accuracy and efficacy of these purchasing behaviours for early cancer detection…". It is not clear to me how you will evaluate this model inside the present study. This will probably require a prospective intervention study.

Personally, I would be very interested in the results of your first secondary aim: "… assess the predictive value of purchasing behaviours in the early detection of these cancers…". Will this be high enough to make public interventions worth wile?

4. How many GP practices will you need to include? I assume they will be recruited through your national GP research networks. If this does not yield enough cases, you will recruit from NHS cancer clinics. Are such clinics also committed to participate in research, so that they have the relevant resources?

5. On p. 11 you describe the data sources available. Suddenly, for the first time you mention the participants' eating habits. I presume that these are included in the loyalty card registrations. How will you make use of this information?

6. On p. 12 you describe data Extraction from the High Street Retailers. You say: "… we will request up to six years in total of back-dated purchase data, depending on the amount of data available." It is unclear to me what this dependence means.

7. Further down on p. 12 you write: "High street retailers will then generate a dataset for all consenting individuals and will share the past purchase data with the CLOCS-2 team using participants' past purchasing data prior to the date of recruitment…". How have you managed to get the retailers to do this job for you? Will it need to be made at each local retailer, og centrally by Boots and Tesco?

8. On p. 14 you say that "the remaining six types of cancer … are being piloted". This fact has not been mentioned before in the protocol, where you just say that CLOCS-2 aims to validate the findings from CLOCS-1 across ovarian cancer as well as nine additional cancers. I think you should inform about the piloting and give a rationale for this already in the Introduction.

9. Will some eligible patients not be able to participate because they do not have the digital skills that the study requires? If so, this should be mentioned as a limitation.

10. It is unclear to me how your controls will be recruited. Is this through the Be Part of Research'? Please provide a closer description, as foreign readers are not familiar with this organisation.

11. There is a printing error on p. 7, line 22: indigestion tables should be indigestion tablets.

VERSION 1 - AUTHOR RESPONSE

Reviewer: 1
Prof. Hans Thulesius, Lund University
Comments to the Author:
The only thing I miss in this protocol paper is the use of STROBE and/or RECORD guidelines checklist.

Thank you for your feedback. We have completed the checklist and included the STROBE guidelines in the protocol (Supplementary S1). Some items are addressed in the protocol, while others will be fully detailed in the final report.

Reviewer: 2
Prof. Mette Brekke, University of Oslo
Comments to the Author:
Thank you for the opportunity to review the protocol for this interesting, elaborate and well-planned study.
I have focused on the presentation of the protocol, not on the design of the study as such. That means: Is the protocol clear? Is something missing? Is the rationale for the study clearly described?
This is a complex and elaborate study, involving retailers, GPs and other healthcare providers, as well as patients and healthy controls. It builds on a similar, but smaller study which the research team has already carried out. All in all, the protocol describes the study well – the rationale, the aim(s), the methods, the analyses, strengths and

weaknesses. The dates for the study are included – recruitment of participants started in February 2026 (as the study is not dependent upon the publication of the protocol). Acceptance from Ethics committee as well as funding sources are revealed.

My comments below mainly refer to questions around some unclarities:
1. You aim to recruit 1 450 participants with one of the cancers of interest (cases) and 1 450 participants without cancer (controls). But you also say: "Controls will be randomly sampled from patients without a cancer diagnosis who meet the matching criteria, typically selecting multiple controls per case…" How does this relate to the consistent numbers of 1 450 in each group?

> *Thank you for this helpful comment. We have clarified the sampling approach in the revised manuscript. The text now specifies that controls will be randomly sampled from participants without a cancer diagnosis who meet the matching criteria, selecting one control per case, ensuring equal numbers (1,450 cases and 1,450 controls) in each group. The text now reads*
>
> ***"Controls will be randomly sampled from participants without a cancer diagnosis who meet the matching criteria, typically selecting one control per case."***
>
> 2. In the framed description of strengths and limitations on p. 4 you say: …" investigate whether routinely collected commercial purchase data can be used to reduce delayed diagnosis of the following cancers…". This may be your ultimate goal, however it is not the aim of the present study, even if one of your secondary aims is to develop theoretical models based upon the results. Further down (page 6) you write: "If successful this study may lead to future interventions…". This seems to be the correct way of presenting your hopes. To show reduced delayed diagnoses would require an intervention study.

Thank you for this helpful comment. In line with this feedback and the editor's earlier comments, we have revised this section to ensure it focuses solely on some of the strengths and limitations of the study design and removed any statements referring to reducing delayed diagnosis or future impact.

3. This comment relates to the one above. On p. 7 you describe your secondary aims and write that one of these are to "Develop and evaluate a predictive model to determine the accuracy and efficacy of these purchasing behaviours for early cancer detection…". It is not clear to me how you will evaluate this model inside the present study. This will probably require a prospective intervention study.
Personally, I would be very interested in the results of your first secondary aim: "…

assess the predictive value of purchasing behaviours in the early detection of these cancers…". Will this be high enough to make public interventions worth wile?

Thank you for your comment. We have clarified the wording in the manuscript and removed "evaluate" from the secondary aim. The current study (CLOCS-2) focuses on developing a predictive model to assess the potential value of purchasing behaviours for early cancer detection. Formal evaluation of the model's accuracy and efficacy will be conducted in a future prospective study such as CLOCS-3.

4. How many GP practices will you need to include? I assume they will be recruited through your national GP research networks. If this does not yield enough cases, you will recruit from NHS cancer clinics. Are such clinics also committed to participate in research, so that they have the relevant resources?

Thank you for this comment. Recruitment will be monitored continuously throughout the study to ensure targets are met. GP practices will be recruited through established national GP research networks, as outlined in the manuscript, and additional sites can be brought on if required to increase recruitment. Recruitment from NHS cancer clinics will be supported by existing research infrastructure, and we have investigators within the study team who are based in these settings and can facilitate site engagement and delivery. Participating sites will therefore have the necessary resources and experience to support study delivery.

5. On p. 11 you describe the data sources available. Suddenly, for the first time you mention the participants' eating habits. I presume that these are included in the loyalty card registrations. How will you make use of this information?

Thank you for this helpful comment. Eating habits data, captured through loyalty card purchases and the health questionnaire, will be used to identify dietary risk factors associated with specific cancers and to examine changes in dietary behaviours in relation to potential symptoms. This will support model development by allowing comparison between purchasing patterns and self-reported dietary information. We have added a clarifying sentence to the manuscript to reflect this

'Eating habits will be used to identify dietary risk factors for specific cancers and to examine changes in dietary behaviour in response to potential cancer symptoms.'

6. On p. 12 you describe data Extraction from the High Street Retailers. You say: "... we will request up to six years in total of back-dated purchase data, depending on the amount of data available." It is unclear to me what this dependence means.

Thank you for pointing this out. We have clarified that while we will request up to six years of back-dated purchase data, the exact amount available for each participant depends on how long they have held their loyalty card. Participants who have held a card for fewer than six years will have correspondingly shorter purchase histories.

The text now reads ***'For both high street retailers, we will request up to six years in total of back-dated purchase data. The exact amount of data available for each participant will depend on how long they have had their loyalty card.'***

7. Further down on p. 12 you write: "will share the past purchase data with the CLOCS-2 team using participants' past purchasing data prior to the date of recruitment...". How have you managed to get the retailers to do this job for you? Will it need to be made at each local retailer, og centrally by Boots and Tesco?

Thank you for this question. The provision of purchase data by high street retailers is covered under established data sharing agreements with the study team. Data extraction will be conducted centrally by the retailers rather than at individual local stores. We have clarified this in the manuscript ***'High street retailers will then centrally generate a dataset for all consenting individuals, in accordance with data sharing agreements, and will share participants' past purchase data with the CLOCS-2 team for the period prior to the date of recruitment.'***

8. On p. 14 you say that "the remaining six types of cancer ... are being piloted". This fact has not been mentioned before in the protocol, where you just say that CLOCS-2 aims to validate the findings from CLOCS-1 across ovarian cancer as well as nine additional cancers. I think you should inform about the piloting and give a rationale for this already in the Introduction.

Thank you for this comment. We have revised the introduction to clarify the study design and added the following text ***'A phased approach will be used. Four cancers (oesophageal, colorectal (bowel), pancreatic, and ovarian), for which symptom profiles and patient pathways are better characterised, will be prioritised for full evaluation. The remaining six cancers will initially be piloted, as there is greater uncertainty regarding symptom presentation and the feasibility of recruitment within these groups. The pilot phase will inform the feasibility of wider inclusion of these cancers in the study.'***

9. Will some eligible patients not be able to participate because they do not have the digital skills that the study requires? If so, this should be mentioned as a limitation.

Thank you for this comment. We have added a limitation noting that the study requires participants to complete all procedures digitally. Participants with limited digital skills may be less able to participate, which could introduce potential selection bias.

Finally, as all study procedures (including consent, questionnaire completion, and submission of loyalty card details) are conducted digitally, individuals with limited digital skills may find it difficult to participate. While some participants may be able to receive assistance, those without access to such support may be less likely to take part, which could introduce selection bias.

The text now reads ***'Finally, as all study procedures (including consent, questionnaire completion, and submission of loyalty card details) are conducted digitally, individuals with limited digital skills may find it difficult to participate. While some participants may be able to receive assistance, those without access to such support may be less likely to take part, which could introduce selection bias.'***

10. It is unclear to me how your controls will be recruited. Is this through the Be Part of Research'? Please provide a closer description, as foreign readers are not familiar with this organisation.
Thank for your comment. Both cases and controls will be recruited through Be Part of Research. As this is a volunteer-based recruitment service, we anticipate that a substantial proportion of controls will be recruited via this route. To clarify this, we have revised the manuscript and added further details. The text now reads ***'Participants (cases and controls) will be recruited across the UK through individual GP practices and Be Part of Research. Additional cases will be recruited through NHS cancer clinics.'***

We have also added extra information about Be Part of Research ***'Be Part of Research is a UK-based participant recruitment service run by the National Institute for Health and Care Research (NIHR), which is open to anyone living in the UK. Registered volunteers provide demographic and health information and can indicate the types of studies they are interested in. The platform then pre-screens individuals based on this information and their stated interests to identify those who may be eligible for specific studies. Individuals who meet these criteria may be contacted by email with information about the CLOCS-2. All respondents will undergo further screening against the study's inclusion and exclusion criteria prior to enrolment. '***

11. There is a printing error on p. 7, line 22: indigestion tables should be indigestion tablets.

Thank you for highlighting this error. We have corrected ***"indigestion tables" to "indigestion tablets".***
Reviewer: 1
If you have selected 'Yes' above, please provide details of any competing interests.: Not applicable

Reviewer: 2
If you have selected 'Yes' above, please provide details of any competing interests.: Not applicable
